# Ecological pattern of microalgal communities and associated risks in coastal ecosystems

**DOI:** 10.1093/ismeco/ycaf109

**Published:** 2025-07-02

**Authors:** Li Zhang, Anqi Xiong, Changchao Li, Xintong Liu, Xiaohua Zhang, Shihao Gong, Meng Yan, Xian Qin, Yang Liu, Zhangxi Hu, James Kar-Hei Fang, Huanfeng Duan, Hongbin Liu, Leo L Chan, Ling N Jin

**Affiliations:** Department of Civil and Environmental Engineering, The Hong Kong Polytechnic University, Hung Hom, Kowloon, Hong Kong SAR 999077, China; Department of Civil and Environmental Engineering, The Hong Kong Polytechnic University, Hung Hom, Kowloon, Hong Kong SAR 999077, China; Department of Civil and Environmental Engineering, The Hong Kong Polytechnic University, Hung Hom, Kowloon, Hong Kong SAR 999077, China; Department of Civil and Environmental Engineering, The Hong Kong Polytechnic University, Hung Hom, Kowloon, Hong Kong SAR 999077, China; Department of Civil and Environmental Engineering, The Hong Kong Polytechnic University, Hung Hom, Kowloon, Hong Kong SAR 999077, China; Department of Civil and Environmental Engineering, The Hong Kong Polytechnic University, Hung Hom, Kowloon, Hong Kong SAR 999077, China; State Key Laboratory of Marine Pollution, City University of Hong Kong, Kowloon Tong, Kowloon, Hong Kong SAR 999077, China; Department of Chemistry, City University of Hong Kong, Kowloon Tong, Kowloon, Hong Kong SAR 999077, China; State Key Laboratory of Marine Pollution, City University of Hong Kong, Kowloon Tong, Kowloon, Hong Kong SAR 999077, China; Key Laboratory of Tropical Marine Bio-resources and Ecology, Guangdong Provincial Key Laboratory of Applied Marine Biology, South China Sea Institute of Oceanology, Chinese Academy of Sciences, Guangzhou, Guangdong 510301, China; Institution of South China Sea Ecology and Environmental Engineering, Chinese Academy of Sciences, Guangzhou, Guangdong 510301, China; College of Fisheries, Guangdong Ocean University, Zhanjiang, Guangdong 524088, China; State Key Laboratory of Marine Pollution, City University of Hong Kong, Kowloon Tong, Kowloon, Hong Kong SAR 999077, China; Department of Food Science and Nutrition, The Hong Kong Polytechnic University, Hung Hom, Kowloon, Hong Kong SAR 999077, China; Research Centre for Nature-based Urban Infrastructure Solutions, Research Institute for Future Food, The Hong Kong Polytechnic University, Hung Hom, Kowloon, Hong Kong SAR 999077, China; Department of Civil and Environmental Engineering, The Hong Kong Polytechnic University, Hung Hom, Kowloon, Hong Kong SAR 999077, China; State Key Laboratory of Marine Pollution, City University of Hong Kong, Kowloon Tong, Kowloon, Hong Kong SAR 999077, China; Department of Ocean Science, The Hong Kong University of Science and Technology, Clear Water Bay, N.T., Hong Kong SAR 999077, China; Hong Kong Branch of Southern Marine Science & Engineering Guangdong Laboratory (Guangzhou), The Hong Kong University of Science and Technology, Clear Water Bay, N.T., Hong Kong SAR 999077, China; State Key Laboratory of Marine Pollution, City University of Hong Kong, Kowloon Tong, Kowloon, Hong Kong SAR 999077, China; Department of Biomedical Sciences, City University of Hong Kong, Kowloon Tong, Kowloon, Hong Kong SAR 999077, China; Department of Civil and Environmental Engineering, The Hong Kong Polytechnic University, Hung Hom, Kowloon, Hong Kong SAR 999077, China; State Key Laboratory of Marine Pollution, City University of Hong Kong, Kowloon Tong, Kowloon, Hong Kong SAR 999077, China; Research Centre for Nature-based Urban Infrastructure Solutions, Research Institute for Future Food, The Hong Kong Polytechnic University, Hung Hom, Kowloon, Hong Kong SAR 999077, China; Department of Health Technology and Informatics; Mental Health Research Centre, The Hong Kong Polytechnic University, Hung Hom, Kowloon, Hong Kong SAR 999077, China; The Hong Kong Polytechnic University Shenzhen Research Institute, Shenzhen 518057, Guangdong, China

**Keywords:** phytoplankton community, ecological pattern, assembly process, deterministic process, toxigenic algae, lipophilic algal toxin

## Abstract

Eukaryotic harmful and toxic microalgae, along with their derived toxins, pose significant threats to seafood safety, human health, and marine ecosystems. Here, we developed a novel full-length 18S rRNA database for harmful and toxic microalgae and combined metabarcoding with toxin analyses to investigate the ecological patterns of phytoplankton communities and the underlying mechanism of associated toxic microalgae risks. We identified 79 harmful and toxic species in Hong Kong’s coastal waters, with dinoflagellates and diatoms representing the majority of toxic and harmful taxa, respectively. Distinct seasonal succession patterns were observed in phytoplankton communities, driven by different ecological assembly processes. Deterministic processes dominated during the dry season, correlating with elevated toxic microalgae abundance and temperature stress. Seasonal shifts in temperature played a pivotal role in shaping toxic algal communities. The dominance of dinoflagellates, particularly *Alexandrium* spp., *Dinophysis* spp., *Prorocentrum* spp., and *Karenia* spp., during the dry season was consistent with elevated toxin concentrations. These toxin profiles highlight the heightened risk in a warming climate, where the prevalence and impacts of toxigenic algae are expected to intensify.

## Introduction

Harmful algal blooms (HABs) have become more widespread, intense, and frequent in coastal areas over the past few decades [1]. These blooms are often caused by microalgae capable of producing toxins that can lead to fish mortality, shellfish poisoning, and even severe human illnesses when contaminated seafood is consumed [2]. To date, >300 phycotoxins and their derivatives have been identified, with ~90% being lipophilic algal toxins (LATs). These toxins are responsible for numerous seafood poisoning incidents reported worldwide [3–8]. According to the Global HAB Status Report 2021 [9], the prevalence of toxin-producing species such as *Dinophysis* spp., *Alexandrium* spp., and *Pseudo-nitzschia* spp. has increased significantly over the past three decades. In addition to toxic species, nontoxic microalgae can form high-biomass blooms that cause severe ecological damage by clogging the gills of aquatic organisms or depleting oxygen levels, leading to hypoxia or anoxia in the surrounding water [10]. Together, these phenomena have caused extensive socioeconomic losses, including mass fish mortality events and the closure of fisheries [11].

Understanding the distribution patterns and the relative roles of deterministic and stochastic processes in shaping microalgal communities across spatiotemporal scales is crucial to predicting how these communities respond to environmental changes. Deterministic processes, driven by environmental filtering and species interactions, suggest that the selection is the primary force shaping microbiomes, while stochastic processes emphasize the roles of dispersal events and random drift [12]. Environmental stressors, such as temperature, salinity, and nutrient availability, are key drivers of deterministic assembly in both macroorganisms and microorganisms [13–16]. However, the regulatory factors often vary among causative species [17–19]. Stakeholders, including policymakers and environmental managers, demand predictive models that integrate environmental drivers with real-time monitoring to anticipate HABs, as well as detailed toxin profiles to assess seafood safety risks and guide public advisories. Addressing these needs requires a comprehensive understanding of the taxonomic diversity of toxigenic algae and the environmental factors influencing toxin production, enabling effective governance and mitigation strategies under changing climatic conditions.

Situated at the transition between the estuarine Pearl River Estuary (PRE) and the open South China Sea, Hong Kong’s coastal waters have been significantly impacted by HABs for decades, with 1817 HAB incidents recorded to date. Although most documented causative species have been nontoxic [20], toxigenic algal species, such as *Dinophysis*, and associated toxins like pectenotoxins (PTXs) can exert covert but persistent impacts on marine ecosystems, even in the absence of observable blooms. Species such as *Coolia*, *Fukuyoa,* and *Amphidinium* in Hong Kong waters have been reported to induce hemolysis in marine fish [21]. This underscores the need for expanded monitoring and research to address not only episodic bloom events but also the ongoing presence and impacts of toxigenic species and their toxins in marine ecosystems.

Emerging methodologies like metabarcoding offer significant advantages for early detection of HAB species and risk assessment, providing greater sensitivity and specificity compared to traditional time-intensive, morphology-based approaches [22]. Many microalgal species are difficult to distinguish morphologically due to their small size [23, 24]. While the accuracy of DNA metabarcoding depends on the quality of reference databases, no dedicated sequence database for harmful and toxic microalgae currently exists.

In this study, we developed a novel and comprehensive full-length 18S rRNA database for harmful and toxic microalgae (HTMaDB) and combined metabarcoding with LAT analyses across a large sample size, providing full spatiotemporal coverage of Hong Kong waters. This approach aimed to (1) investigate the ecological patterns of coastal microalgal communities, (2) uncover the underlying mechanisms driving the assembly processes that control the spatiotemporal dynamics of these communities, and (3) identify potential causative organisms of targeted LATs.

## Materials and methods

### Marine sampling campaign

Marine sampling campaign was conducted along the coastal line of Hong Kong to investigate harmful and toxic species and algal toxins in both wet (August 2022) and dry (February 2023) seasons (Fig. S1). We collected seawater from 36 sampling sites of Hong Kong coastal ecosystems. Surface seawater samples were collected using stainless-steel buckets and were transferred to 1-L polypropylene bottles. The bottles were covered with foil and kept in a portable icy incubator below 4°C during transportation to the laboratory. At each sampling location, a total of 5 L of seawater samples were collected for the analysis of LATs (1 L), nutrients (1 L), phytoplankton density (1 L), and phytoplankton communities (2 L). Three bottles of Milli-Q water were also transported from the laboratory to the field site and then returned to the laboratory. These bottles served as field blanks to ensure negligible contamination. The samples were stored in a 4°C fridge, and all samples were analyzed within 1 month. Salinity, temperature, pH, and dissolved oxygen (DO) were measured in situ using the YSI Professional Plus Quatro water quality meter (YSI Incorporated, Yellow Springs, OH, USA). Solar radiance was measured via SMART SENSOR digital Lux meter AS803 (IntelliSearch, Shenzhen, China). The concentrations of nutrients, including NH_4_^+^, NO_2_^−^, NO_3_^−^, SiO_3_^2−^, and PO_4_^3−^, were determined in the laboratory using automated wet chemistry analyzer (Skalar SAN^++^, Netherlands) (Table S1).

### Construction of harmful and toxic microalgae database

**Figure 1:**
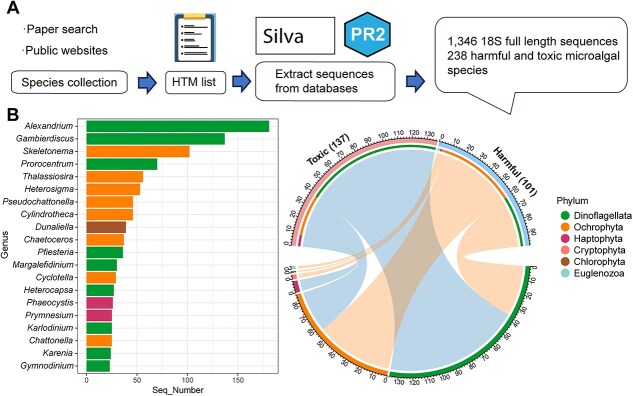
Flowchart illustrating the construction of the 18S sequence database (A). Top 20 genera ranked by sequence count (left) and the taxonomic composition at the phylum level (right) in the constructed HTMaDB (B).

Harmful and toxic algal species were collected based on the IOC-UNESCO Taxonomic Reference List of Harmful Micro Algae and nearly 200 related articles (Supplementary Excel Table 1). Toxic algae were defined as species capable of producing toxins, while harmful algae were defined as nontoxic species that form blooms causing ecological damage. The compiled species information is summarized in Supplementary Excel Table 2. To construct a comprehensive reference database, we targeted species with scientifically accepted names, following the taxonomic framework provided by AlgaeBase to ensure consistency and accuracy in species nomenclature. Using these validated names, a total of 1346 full-length 18S rRNA sequences were retrieved from the Silva 138.1 SSU [25] and PR2 5.0.0 SSU Ref databases [26]. Taxonomic classifications were primarily based on the Silva database, while inconsistencies in species and strain levels were manually resolved by cross-referencing to AlgaeBase [27]. Detailed sequence IDs and taxonomic compositions are provided in Supplementary Excel Table 3, and the entire database construction process is illustrated in Fig. 1A. The resulting harmful and toxic microalgae database, HTMaDB, is available in fasta format and has been made publicly accessible on GitHub (https://github.com/lisse0321/HTMaDB).

A total of 238 eukaryotic harmful and toxic microalgae species were collected (Supplementary Excel Tables 4 and 5). At the phylum level, toxic microalgae encompass dinoflagellates (99), Ochrophyta (30), and Haptophyta (8), whereas harmful microalgae consist of dinoflagellates (37), Ochrophyta (56), Cryptophyta (3), Chlorophyta (1), and Euglenozoa (2) (Fig. 1B). This indicates that dinoflagellates are the dominant contributor of toxins, whereas harmful algal blooms are primarily caused by Ochrophyta. The sequence number of top 20 genera is shown in Fig. 1C.

### DNA extraction, sequencing, and bioinformatic analysis

For the extraction of total algal DNA, seawater was filtered through a 0.45-μm mixed cellulose ester membrane (HAWP04700; Merck Millipore, Burlington, MA, USA). The filters were then stored at −80°C until DNA extraction. Total DNA was extracted from the filters using FastSpinKit for soil (MP Biomedicals, Santa Ana, CA, USA) following the manufacturer’s instructions. Briefly, the membranes were cut into small pieces with sterile scissors and transferred to Lysing Matrix E tubes. Homogenization was performed using the FastPrep instrument for 40 s at a speed setting of 6.0 m s^−1^ with 978 μl sodium phosphate buffer and 122 μl MT buffer to mechanically disrupt algal cell walls and release nucleic acids into the protective buffer. The supernatant was transferred to a clean 2-ml microcentrifuge tube after centrifugation to pellet debris. To separate the solubilized nucleic acids from cellular debris, 250 μl of protein precipitation solution was added, and the mixture was centrifuged again, with the resulting supernatant transferred to a clean 15-ml tube. Flocculated proteins were removed, and 1 ml of binding matrix solution was added to bind nucleic acids. The DNA solution was then transferred to a spin filter tube with an empty catch tube. The prepared SEWS-M solution was added to wash the spin filter, removing impurities by centrifuging through the filter while the purified DNA is still bound to the silica. After air-drying the spin filter for 5 min at room temperature to remove residual ethanol, the binding matrix was resuspended in 100 μl elution buffer. The purified DNA solution was recovered via centrifugation. Finally, the concentration of the extracted DNA was measured using a NanoDrop One Microvolume UV–Vis Spectrophotometer (Thermo Fisher Scientific, Waltham, MA, USA).

The 18S rRNA V9 region was amplified using the universal eukaryotic primers 1380F (5′-CCCTGCCHTTTGTACACAC-3′) and 1510R (5′-CCTTCYGCAGGTTCACCTAC-3′) [28]. The resulting libraries were sequenced on an Illumina HiSeq2500 platform. Paired-end reads were merged using USEARCH v10.0.240 [29], followed by primer trimming and quality control performed with VSEARCH v2.15 [30]*.* Potential chimeric sequences were identified and removed using the chimera search module in USEARCH, with the Silva_18s_v123 reference database used as a reference. Clean amplicons were denoised into amplicon sequence variants (ASVs) in *de novo* mode [31, 32]. During the denoising process, sequencing errors were corrected, and singletons were excluded to minimize noise and improve reliability. Taxonomic assignments of ASVs were performed against the Silva Database (Release 123) [25]. To evaluate sample saturation, a rarefaction curve was generated using the picante and vegan function packages in R (version 4.2.1), as shown in Fig. S2.

### Sample preparation and instrumental analysis for LATs

For the extraction of LATs, seawater samples were extracted and purified using Oasis HLB cartridges (200 mg, 6 ml; Waters, Medford, MA, USA), and the target analytes were subsequently eluted using ammonium hydroxide/methanol (3:1000, v:v) three times. The final extracts were evaporated until dry under a gentle nitrogen stream and reconstituted with methanol. Samples were finally kept at −20°C until further analysis. Details can be found in Section S1 of the Supporting Information (SI). The standards of gymnodium (GYM), okadaic acid (OA), pectenotoxin-2 (PTX-2), dinophysistoxin-1 and -2 (DTX-1 and -2), and azaspiracid-1 and -2 (AZA-1 and -2) were purchased from the National Research Council, Institute for Marine Biosciences (Halifax, Nova Scotia, Canada). The properties of algal toxins are shown in Table S2. The standards were stored at −20°C. Details regarding the standards, reagents, and solvents are provided in Section S2 of SI.

Separation of the seven LATs in seawater samples was performed using an ExionLC UHPLC system (Sciex, Foster City, CA, USA), equipped with an ACQUITY UPLC BEH C18 Column (2.1 mm × 50 mm, particle size: 1.7 μm; Waters). A Sciex 6500+ Liquid chromatography/Electrospray Ionization–QTrap Mass Spectrometer (6500+ QTRAP mass spectrometer; Sciex, Foster City, CA, USA) equipped with an ESI interface was used in MS analysis; details can be found in Section S3 and Tables S3 and S4. The QA/QC measures involved checking for method limits of detection (LODs), quantification (LOQs), calibration curves, and procedure blanks. Each sample was extracted and analyzed in duplicate, and for every batch of real samples, procedural blank (*n* = 2) and blank-spiked recoveries (*n* = 2) were also included.

To determine the LODs and LOQs for LATs, we conducted the same extraction procedure with real environment samples on 11 blank samples. The standard deviation of the signals was calculated; LOD and LOQ can be estimated to 3 times and 10 times of the standard deviation, respectively (Table S5). Calibration curves for the seven LATs had concentrations (seven data points) with correlation coefficients ranging from 0.9977 to 0.9999 (Table S5). Recoveries of the seven LATs varied from 52.9% to 92.5% in seawater and from 89.3% to 116.3% in suspended particulate matter (SPM) samples (Table S5). Importantly, none of the seven target LATs were found in procedure blanks. The reported LAT concentrations were not surrogate recovery corrected.

### Data and statistical analyses

The spatial pattern of phytoplankton community structure was evaluated using principal coordinate analysis (PCoA) based on Bray–Curtis dissimilarity with the “vegan” package in R (version 4.2.1). With ASV relative abundance data, Bray–Curtis distances between samples were calculated. PCoA was performed to reduce the dimensionality of the distance matrix and visualize maximally the similarities or dissimilarities between samples. Differences in the community composition between wet and dry seasons were tested with the permutational multivariate analysis of variance (PERMANOVA). Richness is defined as the total number of unique ASVs per sample. Alpha diversity, which incorporates both richness and evenness, was quantified using the Shannon diversity index. These indices were calculated using the “vegan” package in R.

To understand the mechanisms controlling phytoplankton community patterns, we evaluated the relative roles of stochastic and deterministic processes during the phytoplankton community assembly in different seasons using the index of modified stochasticity ratio (MST) with the “NST” package in R [33]. First, null expectation was generated by randomizing community composition across samples while preserving species richness and total abundance. Then, observed communities were compared with null expectation, with more similar or dissimilar compositions attributed to deterministic factors, otherwise stochastic factors [33]. MST quantified this process, with values <0.5 indicating deterministic dominance and >0.5 indicating stochastic dominance [33]. Statistical significance was assessed using the Mann–Whitney *U* test.

The Spearman’s rank correlations between the relative abundance of toxic taxa and environmental variables, including temperature, salinity, pH, DO, and nutrients (NO_3_^−^, NO_2_^−^, NH_4_^+^, SiO_3_^2−^, and PO_4_^3−^), were calculated using the “netET” package in R. Significant correlations were visualized as a network with the “ggraph” package, where nodes represented toxic taxa or environmental variables, and edges represented significant positive or negative correlations. Edge thickness reflected the strength of the correlation, and colors distinguished between positive and negative relationships. The linear regression analysis was employed to reveal the relationship between temperature and the alpha diversity (richness and Shannon index) of the toxic community. The specificity and occupancy of each toxic species were calculated in each season to characterize specialist taxa [34, 35]*.* Specificity is operationally defined as the average abundance of a given species in the samples of season, while occupancy is characterized as the relative frequency with which species occur within the same season*.* Species with specificity and occupancy ≥0.7 were defined as specialist species, which indicated that they were specific to a season [36]*.* Variation partitioning analysis (VPA) [37] was performed with the “varpart” package to determine the relative contribution of environmental variables and co-occurring nontoxic algal taxa to the variation in toxic algal community composition. Environmental variables and nontoxic algal taxa abundances were *z*-score standardized for the analysis. Results from VPA were visualized as a Venn diagram, and the proportion of unexplained variation was reported as the residual fraction.

Significance thresholds for all statistical tests were set at *P* <.05, unless otherwise specified.

## Results and discussion

### Distinct seasonal pattern of phytoplankton community across Hong Kong coastal seawater

**Figure 2:**
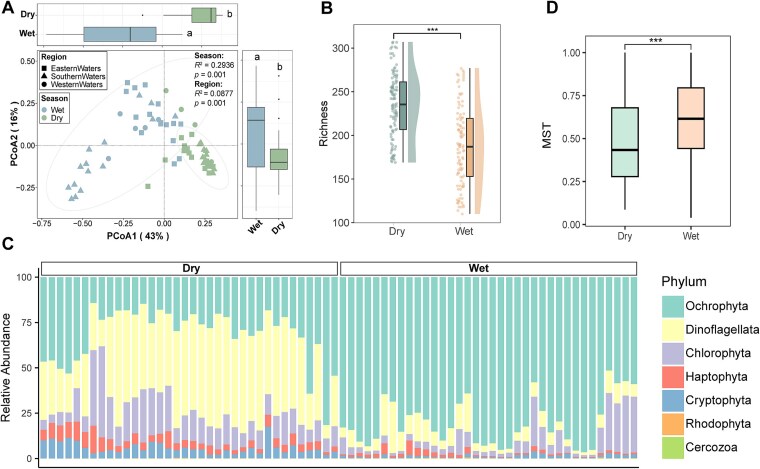
Ecological patterns of the phytoplankton community. Significant seasonal patterns in community structure (A), richness (B), composition (C), and assembly processes based on the modified stochasticity ratio (MST) (D). The MST index uses a threshold of 50% to differentiate between predominantly deterministic assembly (<50%) and predominantly stochastic (>50%) assembly.

Of the 5462 ASVs obtained from 18S rRNA gene amplicon sequencing, 1649 were identified as eukaryotic microalgae. PCoA and PERMANOVA unveiled a notable differentiation in taxonomic compositions between the two sampling seasons compared to between different regions, suggesting that seasonality exerts a stronger influence than spatial factors at a small, localized scale (*R*^2^_season_ = 0.3164 > *R*^2^_region_ = 0.057; Fig. 2A). Taxonomic richness increased during the dry season compared to the wet season (Fig. 2B), and distinct seasonal patterns in phytoplankton composition were observed (Fig. 2C). The eukaryotic phytoplankton were classified into seven phyla: Dinoflagellata, Ochrophyta, Chlorophyta, Haptophyta, Cryptophyta, Rhodophyta, and Cercozoa. Dinoflagellates and Ochrophyta dominated the taxonomic composition, collectively accounting for >70% of the total in both seasons. The relative abundance of dinoflagellates was highest in the dry season, whereas Ochrophyta peaked in the wet season.

Diatoms in the phylum of Ochrophyta and dinoflagellates are two common groups making up the phytoplankton community in Hong Kong coastal waters [38]. The growth of most diatoms depends on the availability of nutrients [39]. Hong Kong waters receive inputs of sewage effluent and storm water discharges all year [40]. The peak of diatom abundance predominantly occurred during the hot and wet summer months, which coincided with high nutrient inputs from the PRE discharge [41]. The discharge containing high level of total inorganic nitrogen outflows and sweeps across most parts of the Hong Kong waters may cause habitat homogenization, resulting in overall decline in biodiversity, which could explain lower richness observed in the wet season [40].

During the dry season, stable hydrological conditions characterized by lower rainfall, minimal freshwater input, and consistent salinity (30–34), creating a predictable environment where niche-based deterministic processes dominate. In contrast, the wet season introduces high environmental stochasticity due possibly to monsoon-driven rainfall, terrestrial runoff, and pulsed nutrient inputs. Salinity fluctuates sharply (18–33), temperatures rise (28–33°C), and turbidity increases, destabilizing niche boundaries. Frequent disturbances, such as typhoons and rapid freshwater influxes, weaken environmental filtering, allowing stochastic processes like dispersal limitation and ecological drift to govern community assembly [42]. This seasonal shift highlights how hydrological stability in the dry season reinforces deterministic selection, while wet-season variability amplifies randomness, shaping distinct phytoplankton dynamics in Hong Kong’s coastal waters.

### Mapping harmful and toxic microalgae using HTMaDB

**Figure 3:**
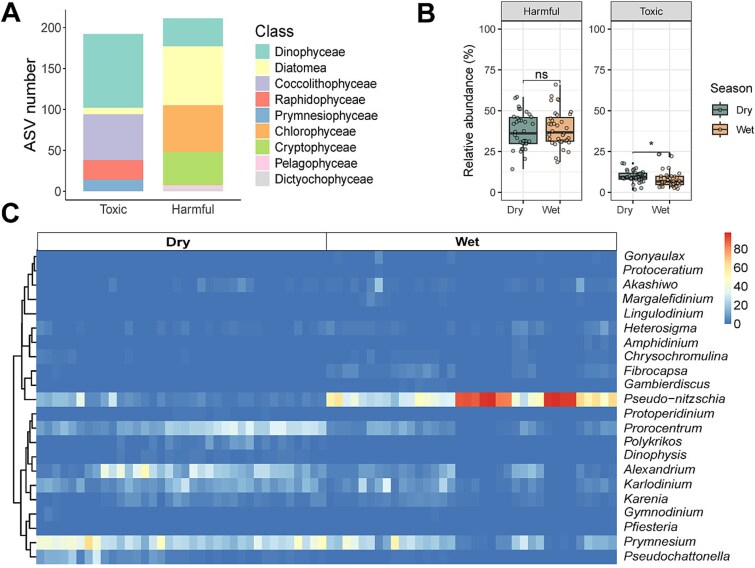
Temporal patterns of harmful and toxic algal communities identified using HTMaDB. The proportion of classified ASV numbers at the class level (A), significantly higher relative abundance of all toxic taxa in the dry season compared to the wet season (B), and temporal distribution of harmful and toxic genera in two seasons (C).

Out of the 1649 eukaryotic microalgal ASVs, 403 were classified as harmful and toxic microalgae in Hong Kong coastal seawater. These ASVs were distributed across nine classes: Dinophyceae, Diatomea, Coccolithophyceae, Raphidophyceae, Prymnesiophyceae, Chlorophyceae, Cryptophyceae, Pelagophyceae, and Dictyochophyceae (Fig. 3A). Among these, Dinophyceae and Diatomea were the dominant classes of toxic and harmful taxa, respectively (Fig. 3A). The relative abundance of 192 toxic ASVs and 211 harmful ASVs displayed different seasonal patterns (Fig. 3B). Toxic algae were more prominent during the dry season, whereas harmful taxa remained relatively stable across seasons. The relative abundance of 22 harmful and toxic algal genera during the wet and dry seasons is shown in Fig. 3C.

Among the 32 identified toxic algal species (Table S6), the diatom genus *Pseudo-nitzschia* exhibited the highest relative abundance, while a greater number of dinoflagellates were associated with the genera *Alexandrium*, *Dinophysis*, *Gymnodinium*, and *Prorocentrum* (Fig. 3C). Additionally, the genera *Chrysochromulina*, *Phaeocystis*, and *Prymnesium* within the phylum Haptophyta were also detected. A total of 47 harmful algal species were identified, most of which are diatoms (Table S6). These harmful and toxic algal taxa have diverse adverse effects on marine ecosystems. For instance, water discoloration and oxygen depletion are often caused by harmful bloom-forming species, particularly diatom taxa and some dinoflagellates.

Toxic algal taxa pose threats to fish and shellfish, often causing mass mortality in aquaculture organisms. Notably, ichthyotoxic dinoflagellates *Margalefidinium polykrikoides* and *Margalefidinium fulvescens* have been linked to such events [43]. *Alexandrium* spp. are major producers of paralytic shellfish poisoning (PSP) toxins [44]. A total of 26 ASVs related to *Alexandrium* in this study were attributed to five species, including *A. andersonii*, *A. hiranoi*, *A. leei*, *A. ostenfeldii*, and *A. tamarense*, all known producers of PSP toxins. *Pseudo-nitzschia* spp., the only known producers of domoic acid, such as *Pseudo-nitzschia australis* and *Pseudo-nitzschia pungens*, can cause amnesic shellfish poisoning [45]. *Dinophysis* spp. and *Prorocentrum* spp. are sources of PTX-2, OA, and its derivative DTX-1, which are responsible for diarrhetic shellfish poisoning [46]. *Karenia mikimotoi* has been responsible for massive die-offs of shellfish, echinoderms, crustaceans, and fish in the coastal waters of many countries. It has been shown to produce several toxins and reactive oxygen species [47]. Additionally, diverse yessotoxin producers, including *Gonyaulax spinifera* and *Protoceratium reticulatum*, were identified in coastal waters. The widespread species *Prymnesium parvum*, predominantly found in coastal waters but also in rivers and marine environments, has been implicated in large-scale fish mortality events globally [48]. Recent studies further suggest that prymnesins, rather than organic micropollutants, exhibit strong *in vitro* neurotoxic effects [49].

### Deterministic processes driven by temperature shaping toxic algal communities

**Figure 4:**
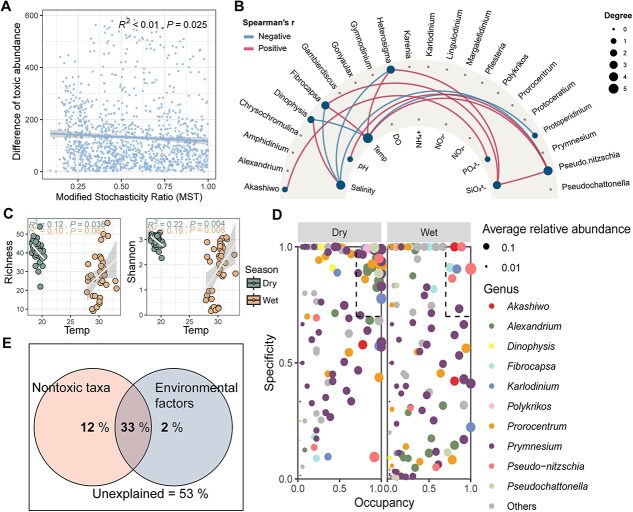
Deterministic processes driven by temperature shape toxic algal communities. Relationships between the stochasticity and the difference in toxic algal abundance (A). Relationships between identified communities and environmental factors. When *P* <.05, a significant correlation is represented by a line. Different colors are used to distinguish between positive and negative correlations (red: positive; blue: negative). The size of the circles represents magnitude of the correlation coefficient, with larger circles indicating a greater impact (B). Relationships between temperature and the alpha diversity (richness and Shannon index) of the toxic algal community (C). The specificity–occupancy plot shows the distribution and specificity of the abundant toxic species with a mean relative abundance >0.01% in each season. Species with specificity and occupancy ≥0.7 are specialist species (D). Variation partitioning analysis showed the effects (%) of co-occurring nontoxic algal taxa and environmental factors (temperature, salinity, pH, DO, and nutrients) on the spatiotemporal distribution of the toxic algal community (E).

The proportion of deterministic process increased with the relative abundance of toxic microalgae from the wet season to the dry season (Fig. 4A). The difference in toxic microalgae abundance was significantly negatively correlated with stochasticity for the toxic algal community (*R*^2^ < 0.01, *P* = .025), suggesting that deterministic processes became more pronounced under higher toxic microalgae stress. This aligns with the theoretical framework in which deterministic assembly intensifies with increasing stress [50]. Under high stress, selective pressures suppress many species while favoring those with greater tolerance, leading to more deterministic community assembly [50].

A correlation analysis revealed significant links toxic algal taxa and environmental variables, including temperature, salinity, pH, and nutrient concentrations (PO_4_^3−^ and SiO_3_^2−^) (Fig. 4B). Temperature emerged as the most influential factor, with five toxic algal taxa—*Dinophysis*, *Fibrocapsa*, *Herterosigma*, *Protoperidinium*, and *Pseudo-nitzschia*—showing positive correlations with temperature, except for *Dinophysis*. This genus was more abundant at cooler temperatures (18–20°C) observed during the dry season, consistent with its optimal growth range of 18–24°C [17]. Rising sea surface temperatures are expected to promote the growth of most toxic algal species, increasing the risk of HABs. However, responses to temperature changes vary across algal groups, species, and strains [51], and short-term responses may obscure longer-term dynamics driven by evolutionary adaptation.

Phytoplankton communities can dynamically adjust their thermal preferences through evolutionary mechanisms [52]. For instance, certain diatom populations have increased their temperature optima by 1°C through metabolic shifts, such as upregulating nitrate utilization genes [53]. This evolutionary process, termed “transcriptional investment or divestment,” reflects shifts in baseline gene expression to match changing thermal regimes [54, 55]. While *Dinophysis* currently dominates cooler seasons, similar adaptations could enable other taxa to expand their thermal niches, reshaping future bloom patterns. However, adaptive capacity varies widely among phytoplankton groups, underscoring the need for cross-taxonomic studies to predict how genetic and metabolic adaptations will redefine HAB risks under persistent warming [56, 57].

Linear regression analysis confirmed that temperature significantly affects the alpha diversity of toxic algal communities (Fig. 4C). Elevated temperature stress was positively correlated with both species richness and the Shannon diversity index, indicating that increased environmental stress fosters toxic algal assemblages. This may be due to the enhanced selection of thermotolerant and opportunistic species. Additionally, negative correlations between three algal taxa and salinity levels were observed, suggesting salinity stress as another environmental driver. Nutrient levels, such as PO_4_^3−^ and SiO_3_^2−^, also promoted the growth of specific taxa like *Pseudo-nitzschia* (diatoms), *Akashiwo* (dinoflagellates), and *Heterosigma* (heterokonts), albeit on a limited scale. Eutrophication in coastal zones could exacerbate the risk of HABs and toxin contamination.

Biotic filtering processes, such as competition, facilitation, and predation, interact with abiotic filtering like environmental stressors to shape toxic algal communities [58]. The occupancy and specificity analysis revealed more keystone toxic species in the dry season (25) than in the wet season (7) (Fig. 4D and Table S7). Dry-season specialists were primarily dinoflagellates, including *Alexandrium*, *Dinophysis*, *Karlodinium*, *Polykrikos*, and *Prorocentrum*, while wet-season specialists were predominantly diatoms like *Pseudo-nitzschia*. This highlights the role of characteristic dinoflagellates in driving the higher algal abundance during the dry season.

VPA showed that 47% of the variation in toxic algal communities could be explained, with nontoxic algal taxa contributing 12%, environmental factors for 2%, and their combined effect 33%; the remaining 53% was unexplained (Fig. 4E). This unexplained variation may reflect complex interactions involving viruses, bacteria, and algae. For example, symbiotic relationships between *Planctomycetaceae* bacteria and diatoms can influence bloom resurgence by providing nutrients and modifying environmental conditions [59, 60].

### Potential causative taxa of targeted LATs

Toxic algal taxa exhibited the same seasonal patterns as targeted LATs (Fig. S3). Of the seven targeted LATs, PTX-2, OA, GYM, and DTX-1 were detected in Hong Kong waters, while DTX-2, AZA-1, and AZA-2 were undetected at all sampling sites (Table S8). Most LATs were found in the dissolved seawater phase, except for PTX-2, which was more prevalent in SPM during the dry season. Within the dissolved phase, PTX-2 and OA emerged as the dominant toxins, collectively accounting for ~80% of the total detected LAT concentrations. These patterns are consistent with those observed in the nearby waters of the Pearl River Estuary [61] and the northern South China Sea [62]. Notably, PTX-2 and OA concentrations surged during the dry season compared to the wet season. DTX-1 was detected exclusively in the dry season, while GYM concentrations showed no significant seasonal fluctuation. *Dinophysis* spp*.*, the sole known producers of PTX-2, exhibit optimal growth at temperatures ranging from 18 to 24°C, which likely explains the higher concentrations of PTX-2 observed during the dry season. In contrast, OA and DTX-1 are more stable in seawater, leading to less pronounced seasonal variations in their distribution when compared to PTX-2. Our recent study [63] shows that these toxins were 1–6 orders of magnitude more potent in cytotoxicity or oxidative stress in Indo-Pacific humpback dolphin and finless porpoise skin fibroblasts than anthropogenic chemicals, resulting in their dominance among known contaminant mixtures eliciting dermal toxic potential in Indo-Pacific marine mammal habitats.

PTXs are synthesized by toxic *Dinophysis* species, which also produce OA and DTXs [64, 65]. In this study, six ASVs related to *Dinophysis* spp. were identified, including *Dinophysis acuminata* and *Dinophysis miles*, both potential producers of PTX-2. These species were observed in Hong Kong waters, although their toxin profiles remain incompletely understood [66]. OA and DTX-1 are primarily produced by *Dinophysis* spp. and epibenthic dinoflagellates of the *Prorocentrum* genus [67]. ASVs linked to *Prorocentrum* species, *Prorocentrum cordatum* and *Prorocentrum rhathymum*, were identified in this study. Notably, *P. rhathymum* has been reported to produce OA. GYM toxins were linked to *Karenia mikimotoi* and *A. ostenfeldii* in our study. Toxic algae related to detected toxins are summarized in Table S9. The relative abundance of toxic algal taxa, particularly *Dinophysis* spp., was significantly higher in the dry season, mirroring the seasonal patterns of corresponding LATs (Fig. S4). These findings provide insights into potential causative organisms of LATs in Hong Kong coastal waters.

Beyond the focus on specific LATs and their direct producers, it is crucial to also consider the broader impacts of other harmful algal species present in Hong Kong’s marine waters (Table S9). For example, species within the *Prorocentrum* group have been linked to fish mortality events, causing hypoxia or anoxia, which can lead to massive fish kills [66]. Additionally, the production of ciguatoxins by *Gambierdiscus scabrosus* poses significant risks. These toxins bioaccumulate through the marine food web, from herbivorous to carnivorous reef fish, ultimately endangering human health through the consumption of contaminated fish [68]. Further research is needed to elucidate the toxin profiles of the prevailing algal species in the region.

## Conclusion

In this study, we investigated the spatiotemporal distribution patterns of microalgal communities and the driving mechanisms behind toxic algal communities during the wet and dry seasons in Hong Kong’s coastal waters. Distinct spatiotemporal patterns in microalgal community dynamics were observed, primarily driven by seasonal shifts in assembly processes. To enhance the identification of harmful and toxic microalgae, we developed a newly curated database, HTMaDB, which serves as a valuable resource for researchers. This database significantly improves the accuracy and efficiency of identification and monitoring strategies for these causative organisms. Overall, we identified 79 harmful and toxic algal taxa, with dinoflagellates and diatoms constituting the majority of toxic and harmful species, respectively. Temperature-driven deterministic processes were found to play a pivotal role in shaping the seasonal dynamics of toxic algal communities. Our findings indicate that rising sea surface temperatures promote the dominance of toxigenic species, thereby amplifying HAB risks in Hong Kong’s coastal ecosystems. While short-term responses highlight temperature-driven shifts in species composition, long-term dynamics may become further intensified as environmental filtering interacts with evolutionary adaptations. To mitigate HAB risks in a warming climate, management strategies must adopt an integrated approach that considers both ecological and evolutionary perspectives.

## Supplementary Material

SI_HTMaDB_11July2025_ycaf109

Supplementary_Harmful_Toxic_Algae_Reflist_ycaf109

## Data Availability

The raw sequencing data from the field-collected seawater samples have been deposited in the NCBI under accession identification numbers PRJNA1105747 (for the 18S rRNA gene amplicon sequencing data).
