# Supplementary material for: Ecological pattern of microalgal communities and associated risks in coastal ecosystems
Source: ISME Commun. 2025 Jul 2;5(1):ycaf109. doi: 10.1093/ismeco/ycaf109 (PMC12404660; doi:10.1093/ismeco/ycaf109)
Supplement: SI_HTMaDB_11July2025_ycaf109 [file si_htmadb_11july2025_ycaf109.pdf]

Supplemental Material

Ecological pattern of microalgal communities and associated risks in coastal ecosystems

Li Zhang[1, #], Anqi Xiong[1, #], Changchao Li[1], Xintong Liu[1], Xiaohua Zhang[1], Shihao Gong[1], Meng Yan[2,3], Xian Qin[2], Yang Liu[4,5], Zhangxi Hu[6], James Kar-Hei Fang [2,7,8], Huanfeng Duan[1], Hongbin Liu[2,9,10], Leo L. Chan[2,11], Ling N. Jin[1,2,8,12,13,*]

[1] Department of Civil and Environmental Engineering, The Hong Kong Polytechnic University, Hung Hom, Kowloon, Hong Kong SAR 999077, China

[2] State Key Laboratory of Marine Pollution, City University of Hong Kong, Kowloon Tong, Kowloon, Hong Kong SAR 999077, China

[3] Department of Chemistry, City University of Hong Kong, Kowloon Tong, Kowloon, Hong Kong SAR 999077, China

[4] Key Laboratory of Tropical Marine Bio-resources and Ecology, Guangdong Provincial Key Laboratory of Applied Marine Biology, South China Sea Institute of Oceanology, Chinese Academy of Sciences, Guangzhou 510301, Guangdong, China.

[5] Institution of South China Sea Ecology and Environmental Engineering, Chinese Academy of Sciences, Guangzhou 510301, Guangdong, China.

[6] College of Fisheries, Guangdong Ocean University, Zhanjiang 524088, Guangdong, China

[7] Department of Food Science and Nutrition, The Hong Kong Polytechnic University, Hung Hom, Kowloon, Hong Kong SAR 999077, China

[8] Research Centre for Nature-based Urban Infrastructure Solutions, Research Institute for Future Food, The Hong Kong Polytechnic University, Hung Hom, Kowloon, Hong Kong SAR 999077, China

[9] Department of Ocean Science, The Hong Kong University of Science and Technology, Clear Water Bay, N.T., Hong Kong SAR 999077, China

[10] Hong Kong Branch of Southern Marine Science & Engineering Guangdong Laboratory (Guangzhou), The Hong Kong University of Science and Technology, Clear Water Bay, N.T., Hong Kong SAR 999077, China

[11] Department of Biomedical Sciences, City University of Hong Kong, Kowloon Tong, Kowloon, Hong Kong SAR 999077, China

[12] Department of Health Technology and Informatics; Mental Health Research Centre, The Hong Kong Polytechnic University, Hung Hom, Kowloon, Hong Kong SAR 999077, China

[13] The Hong Kong Polytechnic University Shenzhen Research Institute, Shenzhen 518057, Guangdong, China

[#]Equal contribution as co-first authors

*Corresponding author

Ling N. Jin

Email: ling.jin@polyu.edu.hk

Tel: +852 2766 6009

FAX: +852 2362 2574

Table of Content

Table S1 *In situ* environmental parameters and nutrients in wet and dry seasons.

Table S2 Molecular weight, molecular formula, and octanol-water partition coefficient of the studied lipophilic algal toxins.

Table S3 Source parameters in negative mode and positive mode.

Table S4 Transitions monitored, dwell times, declustering potentials (DP), entrance potentials (EP), collision cell entrance potentials (CEP) and collisions energies (CE) for the detection of LATs.

Table S5 Limits of detection, limits of quantification, correlation coefficients of the external calibration (r^2), and recoveries of the targeted lipophilic algal toxins.

Table S6 Harmful and toxic microalgae in Hong Kong surface seawater.

Table S7 Specialist species in the dry and wet seasons.

Table S8 The GPS coordinate, concentration of lipophilic algal toxins in dissolved seawater and SPM in sampling location.

Table S9 Toxic algae related to detected toxins.

Section S1 Details about the methods for extracting lipophilic algal toxins.

Section S2 Chemicals and solvents.

Section S3 Details about the instrumental analysis of lipophilic algal toxins.

Figure S1 Sampling locations of surface seawater along the coastline of Hong Kong.

Figure S2 Rarefaction curves of the richness in the wet and dry seasons reach the saturation stage with means and standard errors under 1% - 100%, indicating that the number of samples in our study is sufficient to capture most microorganisms in each season.

Figure S3 Seasonal patterns of targeted LATs.

Figure S4 Seasonal patterns of potential producers.

Table S1 *In situ* environmental parameters and nutrients in wet and dry seasons.

Sampling stations	In-situ parameters				Nutrients ($\mu g \cdot L^{-1}$)				
	Temp (ºC)	Salinity	pH	DO (mg L^{-1})	NO_3^-	NO_2^-	NH_4^+	PO_4^{3-}	SiO_3^{2-}
Wet_S1	30.2	17.5	8.22	5.52	16.7	7.47	42.1	31.5	1487
Wet_S2	33.1	24.9	8.50	2.39	7.35	1.52	55.8	48.2	605
Wet_S3	32.9	27.2	8.35	8.75	6.96	1.33	31.6	45.4	263
Wet_S4	31.3	26.2	8.38	5.55	7.89	1.94	41.7	10.8	478
Wet_S5	30.7	31.9	8.26	5.57	7.33	1.60	33.2	8.26	189
Wet_S6	30.8	32.1	8.18	8.21	7.05	1.23	26.0	5.16	206
Wet_S7	30.9	30.2	8.23	5.83	7.15	1.46	48.5	18.3	157
Wet_S8	30.4	30.5	8.25	5.55	7.33	1.39	40.2	8.18	114
Wet_S9	31.6	31.9	8.19	9.50	6.59	1.06	35.9	39.1	228
Wet_S10	30.4	32.6	8.24	9.60	6.75	1.17	55.5	17.3	121
Wet_S11	30.4	32.5	8.29	9.70	6.76	1.38	42.6	11.2	126
Wet_S12	30.2	31.5	8.24	10.2	6.58	0	47.4	7.05	100
Wet_S13	30.3	32.6	8.25	10.0	6.66	1.18	57.1	10.4	89.2
Wet_S14	30.4	31.6	8.25	10.1	6.79	1.85	47.2	11.8	81.3
Wet_S15	30.7	32.0	8.27	9.80	6.80	1.52	31.1	9.03	95.5
Wet_S16	31.5	32.4	8.06	8.10	6.67	1.17	39.9	16.0	189
Wet_S17	28.1	31.3	8.08	5.56	14.3	10.6	62.0	11.8	129
Wet_S18	27.9	31.1	8.10	5.83	15.1	11.4	52.3	19.9	127

Wet_S19	28.9	30.5	8.17	6.48	11.0	6.33	41.2	17.8	100
Wet_S20	29.0	29.6	8.15	6.39	17.9	14.3	42.9	19.5	114
Wet_S21	28.8	29.7	8.15	6.65	17.3	12.5	38.6	19.0	110
Wet_S22	28.4	28.5	8.06	5.07	43.1	39.0	66.1	17.9	250
Wet_S23	29.0	27.3	8.14	6.00	47.4	43.1	75.8	34.9	311
Wet_S24	29.3	24.5	7.96	4.78	86.4	85.2	79.2	64.6	642
Wet_S25	28.1	28.2	7.95	3.74	46.7	42.9	130	49.6	447
Wet_S26	28.1	27.3	7.74	3.27	66.7	64.6	2101	103.0	635
Wet_S27	28.6	28.2	7.97	5.96	39.9	36.4	120	17.1	380
Wet_S28	29.3	28.0	8.15	5.95	113	115	12.7	77.5	864
Wet_S29	29.6	27.9	8.31	7.80	34.9	29.0	99.6	42.7	306
Wet_S30	30.1	28.2	8.31	7.17	23.9	20.1	66.9	47.8	207
Wet_S31	30.1	27.7	8.14	6.67	18.0	13.0	31.1	24.9	143
Wet_S32	30.8	21.3	7.94	5.48	27.1	22.0	77.7	0	170
Wet_S33	30.2	22.1	7.90	5.74	168	175	2.43	86.7	1181
Wet_S34	30.4	23.4	7.96	5.48	122	125	27.6	47.3	660
Wet_S35	30.6	20.3	7.81	5.29	113	114	0	34.9	681
Wet_S36	31.0	18.8	7.88	6.52	149	153	53.4	91.2	992
Dry_S1	19.7	31.8	7.76	6.74	8.12	7.33	85.7	4.80	258
Dry_S2	20.2	31.9	7.82	6.18	3.02	2.40	34.1	3.10	99
Dry_S3	19.6	32.0	7.83	5.91	2.93	1.69	13.1	2.99	105

Dry_S4	19.7	32.1	7.67	6.57	2.30	1.42	13.5	6.36	139
Dry_S5	19.1	32.6	7.90	6.54	2.24	1.56	11.7	8.86	123
Dry_S6	20.1	32.8	8.07	5.92	1.84	0.95	20.5	4.57	87.6
Dry_S7	19.6	32.9	7.98	5.86	1.72	0.42	28.9	5.62	63.4
Dry_S8	18.9	33.0	8.06	5.74	0	0.93	15.8	4.20	63.3
Dry_S9	18.8	33.3	7.92	5.47	2.33	0.93	45.2	2.89	74.6
Dry_S10	20.0	33.6	7.89	5.78	2.53	1.44	34.9	7.61	89.6
Dry_S11	19.5	33.6	7.88	5.72	5.65	4.28	51.9	8.63	113
Dry_S12	19.8	33.9	7.73	5.54	3.82	3.02	26.2	4.91	82.6
Dry_S13	19.5	33.7	7.88	5.87	4.97	3.79	64.5	7.70	108
Dry_S14	19.2	33.9	7.72	5.75	7.29	6.05	50.7	7.67	114
Dry_S15	19.1	33.6	7.83	5.84	6.42	5.42	52.5	7.53	125
Dry_S16	19.3	33.3	7.79	5.99	2.11	1.96	52.3	5.95	96.1
Dry_S17	18.3	33.1	7.86	5.90	6.28	5.69	74.2	6.51	62.4
Dry_S18	18.2	33.4	7.19	5.87	8.62	7.90	85.3	12.0	129
Dry_S19	20.4	34.0	7.78	6.05	3.05	2.32	52.8	5.43	92.6
Dry_S20	18.2	33.3	7.68	6.12	4.84	4.21	43.1	7.84	113
Dry_S21	18.5	33.0	7.85	6.74	2.66	2.01	46.7	5.90	75.0
Dry_S22	19.2	32.7	7.82	6.18	2.79	2.53	125	10.2	69.7
Dry_S23	18.4	33.1	7.72	5.91	4.99	4.26	144	7.87	79.2

Dry_S24	18.7	32.7	8.06	6.57	4.76	4.42	141	16.4	75.5
Dry_S25	18.8	32.5	7.91	6.54	5.61	4.98	226	13.1	74.1
Dry_S26	18.6	33.0	7.87	5.92	7.22	6.12	127	6.36	67.6
Dry_S27	18.9	31.4	7.80	5.86	9.53	8.85	1212	12.7	165
Dry_S28	18.8	32.6	7.87	5.74	6.55	5.59	135	3.81	111
Dry_S29	18.5	32.2	7.78	5.47	4.22	3.50	113	0.12	82.4
Dry_S30	19.1	33.0	7.88	5.78	3.78	3.38	72.0	2.45	75.0
Dry_S31	19.1	33.2	7.86	5.72	4.67	3.92	72.7	1.91	81.8
Dry_S32	18.9	32.9	7.83	5.54	5.17	4.72	72.7	3.18	81.1
Dry_S33	19.6	30.8	7.95	5.87	9.63	8.58	152	10.2	52.1
Dry_S34	19.1	32.9	7.91	5.75	4.27	3.50	45.2	1.28	78.2
Dry_S35	19.7	30.6	7.85	5.84	7.69	6.80	108	9.61	61.9
Dry_S36	19.4	30.4	8.04	5.99	10.8	9.63	135	8.43	76.0

Table S2 Molecular weight, molecular formula, and octanol-water partition coefficient of the studied lipophilic algal toxins.

Compound	CAS number	Molecular Weight ($g \cdot mol^{-1}$)	Molecular Formula	Log K_{ow} [1]
OA	78111-17-8	805.0	$C_{44}H_{68}O_{13}$	5.05
DTX-1	81720-10-7	819.0	$C_{45}H_{70}O_{13}$	6.88
DTX-2	139933-46-3	805.0	$C_{44}H_{68}O_{13}$	5.61
PTX-2	97564-91-5	857.0	$C_{47}H_{70}O_{14}$	6.47
GYM	173792-58-0	507.7	$C_{32}H_{45}NO_4$	6.64
AZA-1	214899-21-5	842.1	$C_{47}H_{71}NO_{12}$	7.54
AZA-2	265996-92-7	856.1	$C_{48}H_{73}NO_{12}$	8.18

Table S3 Source parameters in negative mode and positive mode.

Parameters	Negative mode	Positive mode
Curtain Gas	25	25
Collision Gas	Low	Low
Voltage (V)	-4500	5000
Temperature (℃)	550	550
Gas 1 (psi)	60	60
Gas 2 (psi)	60	60

Table S4 Transitions monitored, dwell times, declustering potentials (DP), entrance potentials (EP), collision cell entrance potentials (CEP) and collisions energies (CE) for the detection of LATs.

Compound	Transitions (m/z)	Time (ms)	DP (V)	EP (V)	CE (V)	CXP (V)	Precursor ion
OA	803.1>255.1	40	140	-10	-60	-20	$[M-H]^-$
	803.1>563.1	40	140	-10	-58	-25	
DTX-2	803.5>255.2	40	140	-10	-60	-38	$[M-H]^-$
	803.5>563.2	40	140	-10	-58	-17	
DTX-1	817.6>255.2	40	140	-10	-60	-15	$[M-H]^-$
	817.6>563.2	40	140	-10	-58	-30	
YTX	570.4>467.2	40	140	-10	-40	-28	$[M-2H]^{2-}$
	570.4>501.8	40	140	-10	-31	-30	

GYM	508.3>490.3	40	140	10	32	26	$[M+H]^+$
	508.3>392.3	40	140	10	46	30	
	508.3>202.4	40	140	10	50	10	
PTX-2	876.6>823.3	40	140	10	34	20	$[M+NH_4]^+$
	876.6>805.3	40	140	10	37	20	
AZA-1	842.5>824.6	40	140	10	43	27	$[M+H]^+$
	842.5>806.2	40	140	10	46	35	
AZA-2	856.7>838.4	40	140	10	41	38	$[M+H]^+$
	856.7>820.5	40	140	10	49	40	

Table S5 Limits of detection, limits of quantification, correlation coefficients of the external calibration (r^2), and recoveries of the targeted lipophilic algal toxins.

LATs	LOD ($pg L^{-1}$)	LOQ ($pg L^{-1}$)	r^2	Recovery Mean (SW)	Recovery Mean (SPM)
PTX-2	2.83	9.43	0.9977	81.2%	116%
AZA-2	0.58	1.94	0.9988	52.9%	91.2%
GYM	1.15	3.83	0.9993	66.3%	89.3%
AZA-1	0.66	2.20	0.9999	56.3%	101%
OA	9.60	32.0	0.9984	84.7%	105%
DTX-1	3.12	10.4	0.9988	63.0%	96.9%
DTX-2	2.54	8.46	0.9980	92.5%	108%

Table S6 Harmful and toxic microalgae in Hong Kong surface seawater.

Phylum	Species	Impacts	Phylum	Species	Impacts
Dinophyta	*Akashiwo sanguinea*	Toxic	Ochrophyta	*Heterosigma akashiwo*	Toxic
	Alexandrium andersonii	Toxic		*Fibrocapsa japonica*	Toxic
	Alexandrium hiranoi	Toxic		*Pseudo-nitzschia australis*	Toxic
	Alexandrium leei	Toxic		*Pseudo-nitzschia cuspidata*	Toxic
	Alexandrium ostenfeldii	Toxic		*Pseudo-nitzschia delicatissima*	Toxic
	Alexandrium tamarense	Toxic		*Pseudochattonella verruculosa*	Toxic
	Amphidinium klebsii	Toxic		*Asterionellopsis glacialis*	Harmful
	Dinophysis acuminata	Toxic		*Aureococcus anophagefferens*	Harmful
	Dinophysis miles	Toxic		*Chaetoceros affinis*	Harmful
	Gambierdiscus scabrosus	Toxic		*Chaetoceros debilis*	Harmful
	Gonyaulax spinifera	Toxic		*Chaetoceros diadema*	Harmful
	Gymnodinium catenatum	Toxic		*Chaetoceros lorenzianus*	Harmful
	Karenia mikimotoi	Toxic		*Chaetoceros pseudocurvisetus*	Harmful
	Karlodinium veneficum	Toxic		*Chaetoceros rostratus*	Harmful
	Lingulodinium polyedra	Toxic		*Chaetoceros simplex*	Harmful
	Margalefidinium fulvescens	Toxic		*Chaetoceros socialis*	Harmful
	Margalefidinium polykrikoides	Toxic		*Chaetoceros tenuissimus*	Harmful
	Pfiesteria piscicida	Toxic		*Chaetoceros throndsenii*	Harmful
	Polykrikos hartmannii	Toxic		*Coscinodiscus radiatus*	Harmful

	Prorocentrum cordatum	Toxic	*Cyclotella meneghiniana*	Harmful
	Prorocentrum rhathymum	Toxic	*Cylindrotheca closterium*	Harmful
	Protoceratium reticulatum	Toxic	*Guinardia flaccida*	Harmful
	Protoperidinium crassipes	Toxic	*Lepidodinium chlorophorum*	Harmful
	Gonyaulax polygramma	Harmful	*Levanderina fissa*	Harmful
	Gymnodinium impudicum	Harmful	*Dictyocha fibula*	Harmful
	Heterocapsa rotundata	Harmful	*Eucampia zodiacus*	Harmful
	Heterocapsa triquetra	Harmful	*Lauderia annulata*	Harmful
	Noctiluca scintillans	Harmful	*Leptocylindrus danicus*	Harmful
	Pseudocochlodinium profundisulcus	Harmful	*Nitzschia longissima*	Harmful
	Prorocentrum triestinum	Harmful	*Paralia sulcata*	Harmful
	Protodinium simplex	Harmful	*Proboscia alata*	Harmful
	Pyrophacus steinii	Harmful	*Skeletonema marinoi*	Harmful
	Tripos furca	Harmful	*Tenuicylindrus belgicus*	Harmful
	Tripos fusus	Harmful	*Thalassiosira allenii*	Harmful
Haptophyta	*Prymnesium parvum*	Toxic	*Thalassiosira curviseriata*	Harmful
	Chrysochromulina leadbeateri	Toxic	*Thalassiosira mala*	Harmful
	Prymnesium polylepis	Toxic	*Thalassiosira pseudonana*	Harmful
Cryptophyta	*Plagioselmis prolonga*	Harmful	*Thalassiosira punctigera*	Harmful
	Teleaulax acuta	Harmful	*Thalassiosira weissflogii*	Harmful
Chlorophyta	*Dunaliella salina*	Harmful		

Table S7 Specialist species in the dry and wet seasons.

No.	Season	OTU	Specificity	Occupancy	Species
1	Wet	ASV_342	1	0.805555556	*Akashiwo sanguinea*
2	Wet	ASV_1576	0.910852713	0.75	*Fibrocapsa japonica*
3	Wet	ASV_292	0.904225352	0.833333333	*Karlodinium veneficum*
4	Wet	ASV_438	0.733333333	0.861111111	*Prymnesium parvum*
5	Wet	ASV_337	1	0.888888889	*Pseudo-nitzschia cuspidata*
6	Wet	ASV_641	0.863829787	0.777777778	*Pseudo-nitzschia cuspidata*
7	Wet	ASV_13	0.905371556	1	*Pseudo-nitzschia delicatissima*
8	Dry	ASV_209	0.951785714	0.944444444	*Alexandrium andersonii*
9	Dry	ASV_634	0.911111111	0.888888889	*Alexandrium andersonii*
10	Dry	ASV_115	0.854388635	0.972222222	*Alexandrium hiranoi*
11	Dry	ASV_153	0.888530928	1	*Alexandrium tamarense*
12	Dry	ASV_525	0.825174825	0.944444444	*Alexandrium tamarense*
13	Dry	ASV_574	0.909482759	0.833333333	*Alexandrium tamarense*
14	Dry	ASV_431	0.893548387	0.833333333	*Alexandrium tamarense*
15	Dry	ASV_726	0.974358974	0.75	*Dinophysis miles*
16	Dry	ASV_701	0.991836735	0.805555556	*Karenia mikimotoi*
17	Dry	ASV_290	0.776990649	1	*Karlodinium veneficum*
18	Dry	ASV_460	0.992025518	0.833333333	*Polykrikos hartmannii*
19	Dry	ASV_503	0.925816024	0.888888889	*Prorocentrum cordatum*
20	Dry	ASV_989	0.860606061	0.805555556	*Prorocentrum cordatum*
21	Dry	ASV_70	0.917433538	1	*Prorocentrum rhathymum*
22	Dry	ASV_117	0.830131827	1	*Prymnesium parvum*
23	Dry	ASV_163	0.937427578	1	*Prymnesium parvum*
24	Dry	ASV_111	0.988690476	1	*Prymnesium parvum*
25	Dry	ASV_353	0.985887097	0.916666667	*Prymnesium parvum*
26	Dry	ASV_173	0.990983607	1	*Prymnesium parvum*
27	Dry	ASV_451	0.882926829	1	*Prymnesium parvum*
28	Dry	ASV_763	0.966292135	0.777777778	*Prymnesium parvum*
29	Dry	ASV_881	0.704761905	0.75	*Prymnesium parvum*
30	Dry	ASV_1178	0.887096774	0.722222222	*Prymnesium parvum*
31	Dry	ASV_308	0.970260223	0.722222222	*Prymnesium polylepis*
32	Dry	ASV_225	0.949579832	0.972222222	*Pseudo-nitzschia australis*

33	Dry	ASV_105	0.995830438	0.916666667	*Pseudochattonella verruculosa*
34	Dry	ASV_254	0.88558952	0.972222222	*Pseudochattonella verruculosa*

Table S8 The GPS coordinate, concentration of lipophilic algal toxins in dissolved seawater and SPM in sampling location.

Sampling location	Sampling area	Longitude	Latitude	Wet season (ng L^{-1})								Dry season (ng L^{-1})							
				Dissolved phase				SPM				Dissolved phase				SPM			
				PTX-2	OA	GYM	DTX-1	PTX-2	OA	GYM	DTX-1	PTX-2	OA	GYM	DTX-1	PTX-2	OA	GYM	DTX-1
S1	*Eastern Waters*	114.218333	22.408611	0.85	0.68	0.18	<LOD	0.16	<LOD	0.01	<LOD	0.31	1.23	1.43	0.03	0.18	0.00	0.03	<LOD
S2	*Eastern Waters*	114.205	22.450556	0.31	1.12	0.31	<LOD	0.06	<LOD	0.01	<LOD	0.69	1.94	1.76	0.04	0.22	0.00	0.05	<LOD
S3	*Eastern Waters*	114.274167	22.46	1.38	0.78	0.18	<LOD	0.07	<LOD	0.01	<LOD	1.01	1.10	0.98	0.05	0.36	0.00	0.07	<LOD
S4	*Eastern Waters*	114.275278	22.434444	1.39	1.02	0.29	<LOD	0.04	<LOD	0.02	<LOD	1.08	1.89	1.68	0.06	0.29	0.00	0.02	<LOD
S5	*Eastern Waters*	114.335	22.476944	1.97	1.03	0.16	<LOD	0.01	<LOD	0.00	<LOD	1.06	1.64	1.39	0.11	0.53	0.00	0.02	<LOD
S6	*Eastern Waters*	114.3071	22.523683	1.56	1.54	0.23	<LOD	0.05	<LOD	0.03	<LOD	5.38	1.60	0.45	0.64	1.89	0.00	0.01	<LOD
S7	*Eastern Waters*	114.3618	22.57305	0.10	0.31	0.01	<LOD	0.04	<LOD	0.02	<LOD	0.90	0.63	0.30	0.10	1.21	0.10	0.01	<LOD
S8	*Eastern Waters*	114.40945	22.48765	0.87	1.51	0.06	<LOD	0.04	<LOD	0.01	<LOD	0.93	0.37	0.26	0.09	1.78	0.11	0.01	<LOD
S9	*Eastern Waters*	114.289444	22.343056	1.13	0.48	0.34	<LOD	0.11	<LOD	0.00	<LOD	0.58	0.57	0.05	0.06	0.63	0.03	0.00	<LOD
S10	*Eastern Waters*	114.296389	22.286111	1.83	0.98	0.46	<LOD	0.00	<LOD	0.00	<LOD	0.15	0.18	0.00	0.03	1.69	0.05	0.02	0.11
S11	*Eastern Waters*	114.3325	22.276111	1.44	0.44	0.74	<LOD	0.28	<LOD	0.01	<LOD	0.41	0.52	0.00	0.04	0.97	0.02	0.00	0.05
S12	*Eastern Waters*	114.343333	22.249722	1.51	0.11	0.51	<LOD	0.20	<LOD	0.01	<LOD	0.24	0.34	0.06	0.02	0.08	0.01	0.00	<LOD
S13	*Eastern Waters*	114.304167	22.289444	0.97	0.46	0.78	<LOD	0.20	<LOD	0.01	<LOD	0.65	0.31	0.18	0.04	0.79	0.01	0.00	<LOD
S14	*Eastern Waters*	114.379722	22.265	1.29	0.31	0.32	<LOD	0.24	<LOD	0.03	<LOD	0.09	0.18	0.09	0.01	1.25	0.02	0.00	<LOD
S15	*Eastern Waters*	114.394167	22.355	1.00	0.39	0.55	<LOD	0.25	<LOD	0.01	<LOD	0.58	0.32	0.00	0.09	1.73	0.04	0.00	<LOD
S16	*Southern Waters*	114.322222	22.366667	1.03	0.62	0.43	<LOD	0.18	<LOD	0.01	<LOD	0.93	0.94	0.11	0.12	0.69	0.03	0.00	<LOD

S17	*Southern Waters*	114.257222	22.234167	0.50	0.17	0.34	<LOD	0.23	<LOD	0.01	<LOD	1.46	0.73	0.13	0.14	0.58	0.03	0.00	<LOD
S18	*Southern Waters*	114.256389	22.205	0.63	0.09	0.14	<LOD	0.02	<LOD	0.00	<LOD	0.51	0.45	0.10	0.09	0.32	0.00	0.00	<LOD
S19	*Southern Waters*	114.286389	22.155278	0.68	0.19	0.16	<LOD	0.14	<LOD	0.00	<LOD	0.02	0.03	0.01	0.00	0.09	0.00	0.00	<LOD
S20	*Southern Waters*	114.175556	22.1875	0.48	0.07	0.08	<LOD	0.03	<LOD	0.00	<LOD	0.64	0.38	0.00	0.06	1.69	0.04	0.00	<LOD
S21	*Southern Waters*	114.189444	22.227222	0.64	0.08	0.13	<LOD	0.03	<LOD	0.00	<LOD	0.93	0.86	0.00	0.00	0.88	0.03	0.00	<LOD
S22	*Southern Waters*	114.130833	22.228056	0.59	0.21	0.14	<LOD	0.05	<LOD	0.01	<LOD	0.88	1.07	0.00	0.00	2.37	0.04	0.00	<LOD
S23	*Southern Waters*	114.073333	22.278333	0.78	0.18	0.09	<LOD	0.12	<LOD	0.00	<LOD	0.18	0.56	0.00	0.00	0.80	0.03	0.00	<LOD
S24	*Southern Waters*	114.055	22.353333	0.21	0.37	0.01	<LOD	0.07	<LOD	0.00	<LOD	0.70	0.67	0.01	0.00	1.56	0.03	0.00	<LOD
S25	*Southern Waters*	114.119722	22.331667	0.19	0.15	0.05	<LOD	0.07	<LOD	0.02	<LOD	0.18	0.37	0.11	0.00	0.89	0.00	0.00	<LOD
S26	*Southern Waters*	114.117222	22.311667	0.55	0.26	0.05	<LOD	0.05	<LOD	0.00	<LOD	0.21	0.34	0.17	0.00	0.84	0.00	0.00	<LOD
S27	*Southern Waters*	114.1525	22.299167	0.38	0.38	0.05	<LOD	0.02	<LOD	0.01	<LOD	1.47	0.58	0.17	0.00	1.14	0.00	0.00	<LOD
S28	*Southern Waters*	114.009722	22.238889	0.25	0.33	0.02	<LOD	0.10	<LOD	0.02	<LOD	0.19	0.40	0.22	0.02	0.37	0.00	0.00	<LOD
S29	*Southern Waters*	114.002778	22.188056	0.2	0.33	0.01	<LOD	0.09	<LOD	0.00	<LOD	0.30	0.33	0.15	0.03	0.82	0.02	0.00	<LOD
S30	*Southern Waters*	113.939444	22.185556	0.11	0.15	0.01	<LOD	0.09	<LOD	0.00	<LOD	0.23	0.31	0.10	0.02	0.57	0.00	0.00	<LOD
S31	*Southern Waters*	113.881389	22.166944	0.55	0.26	0.05	<LOD	0.14	<LOD	0.02	<LOD	0.57	0.47	0.15	0.04	1.15	0.03	0.00	<LOD
S32	*Western Waters*	113.827983	22.224317	0.12	0.15	0.01	<LOD	0.05	<LOD	0.00	<LOD	0.35	0.42	0.12	0.02	4.90	0.00	0.00	<LOD
S33	*Western Waters*	113.975	22.323056	0.18	0.08	0.04	<LOD	0.04	<LOD	0.00	<LOD	0.29	0.71	0.03	0.06	1.14	0.02	0.00	<LOD
S34	*Western Waters*	113.875	22.289722	0.35	0.12	0.02	<LOD	0.05	<LOD	0.00	<LOD	0.35	0.32	0.11	0.01	1.08	0.00	0.00	<LOD
S35	*Western Waters*	113.919722	22.431944	0.2	0.16	0.02	<LOD	0.04	<LOD	0.00	<LOD	0.29	0.79	0.01	0.06	0.74	0.00	0.00	<LOD
S36	*Western Waters*	113.905278	22.361111	0.19	0.12	0.01	<LOD	0.03	<LOD	0.00	<LOD	0.58	0.75	0.00	0.10	1.36	0.00	0.00	<LOD

(<LOD represents below the limit of detection)

Table S9 Toxic algae related to detected toxins.

Phylum	Class	Order	Family	Genus	Species	Toxin	Reference
Dinoflagellate	Dinophyceae	Dinophysiales	Dinophysaceae	*Dinophysis*	*D. acuta*	OA,	[2]
						DTX-2, PTX-2, PYX-2sa	
						OA,	[3]
						DTX-1	
					D. acuminata	OA	[3]
						DTX-1,	[4]
						PTX-2	
					D.caudate	OA, PTX-2, PTX-2sa	[5]
					D. fortii	OA,	
						DTX-1,	[3]
						PTX-2	
					D. miles	OA, DTX-1	[6]
					D. sacculus	OA, DTX-1	[7]
					D. norvegica	OA,	[3]
						DTX-1	
						PTX-2	[8]
					D. mitra	DTX-1	[3]
					D. rotundata	DTX-1	[3]

			D. tripos	DTX-1	[3]
Prorocentrales	Prorocentraceae	*Prorocentrum*	*P. lima*	OA	[9]
				OA, DTX-1	[3]
			P. faustiae	OA, DTX-1	[10]
			P. concavum	OA	[11]
			P. hoffmannianum	OA	[12]
			P. maculosum	OA	[13]
			P. belizeanum	OA	[14]
			P. porosum	OA	[15]
			P. rhathymum	OA	[16]
Gymnodiniales	Kareniaceae	*Karenia*	*K. mikimotoi*	GYM-A	[17]
Gonyaulacales	Ostreopsidaceae	*Alexandrium*	*A. ostenfeldii*	12-methyl GYM	[18]
			(*A. peruvianum*)	GYM, 12-methyl GYM	[19]

Section S1 Details about the methods for extracting lipophilic algal toxins.

For extraction of LATs, all seawater samples were filtered by 0.45 μm glass microfiber filters (GF/A, Whatman Schleicher & Schuell, Maidstone, England) to remove the visible particulate matter and algal cells. SPE was then conducted with Oasis HLB cartridges (200 mg, 6 mL, Waters, Medford, MA, USA) based on the method described in a previous study (Li et al., 2014). After the cartridges were pre-conditioned with 4 mL of ammonium hydroxide/methanol (3:1000, v:v), 4 mL of methanol and 4 mL of deionized water, a total volume of 250 mL of seawater samples was loaded into each cartridge at a flow rate of 1 $mL \cdot min^{-1}$. Then the cartridges were rinsed with 4 mL of methanol/water (15:85, v:v) and dried by centrifuging at 3500 rpm for 2 mins. The extracts were achieved by elution of the cartridge with 3 mL of ammonium hydroxide/methanol (3:1000, v:v) three times. Subsequently, the extracts were evaporated until dry under a gentle nitrogen stream at a temperature of 40°C, reconstituted with 100 μL of methanol, and centrifuged at 3500 rpm for 3 mins before being transferred to an analysis vial.

For extraction of SPM, each filter loaded with suspended particulate matter was cut off in pieces and placed in 50 mL centrifuges. Samples were extracted with 8 mL 26 mM Ammonia-MeOH by 30 mins ultrasonication. The liquid in the centrifuge tube was added to a 5 mL syringe and filtered through a 0.22μm PTFE filter membrane into a 15 mL centrifuge tube. Conduct other 2 extractions with 5 mL 26 mM Ammonia-MeOH separately. The extract was blown down to dryness under a gentle stream of nitrogen. Samples were reconstituted with 100 μL of methanol and filtered through 0.22 μm PTFE filter before being transferred to an analysis vial.

Section S2 Chemicals and solvents.

Acetonitrile and methanol of gradient grade were purchased from Merck (Darmstadt, Germany). Ammonium hydroxide (≥ 25%) of MS grade was purchased from SUPELCO (Bellefonte, PA, USA). Ultrapure water was obtained from a Milli-Q water-purification system (Millipore, Billerica, MA, USA).

Section S3 Details about the instrumental analysis of lipophilic algal toxins.

Separation of the seven lipophilic algal toxins (LATs) in seawater samples was performed using an ExionLC UHPLC system (Sciex, Foster City, CA, USA), equipped with an ACQUITY UPLC BEH C18 Column (2.1 mm × 50 mm, particle size: 1.7 μm, Waters, Medford, MA, USA). The column was kept at 40°C. A gradient mobile phase of ammonia in Milli-Q water (solvent A, pH = 11) and ammonia in acetonitrile/Milli-Q water 90:10 (solvent B, pH = 11) was used. At a flow rate of 0.5 mL min^{-1}, the gradient condition (in a sequence of 5% solvent B) was increased to 45% at 3.5 mins, followed by a linear increase to 55% at 6.5 mins, which finally increased to 100% at 7.5 mins. and after 1 min, the gradient was converted to the original conditions at 9.5 mins, held for 2 mins. The total run time was 11.5 mins. Then, 10 μL was injected for each sample, and sample illumination of the autosampler was turned off during sample analysis.

The MS method was developed based on previous research (Biotoxins 2015, García-Altares et al. 2013), and optimization was conducted to achieve the best sensitivity in 6500+ QTRAP. The instrumental data were acquired and processed using Analyst 1.6.3 software (Sciex, Foster City, CA, USA). For each target compound, two product ions from the precursor ion were monitored for identification, and the more intense one was used for quantification. Together with the retention times, the precursor ion and the two product ions were used to ensure correct peak identification. The quantification of the target compounds in seawater samples was performed with an external standard method.

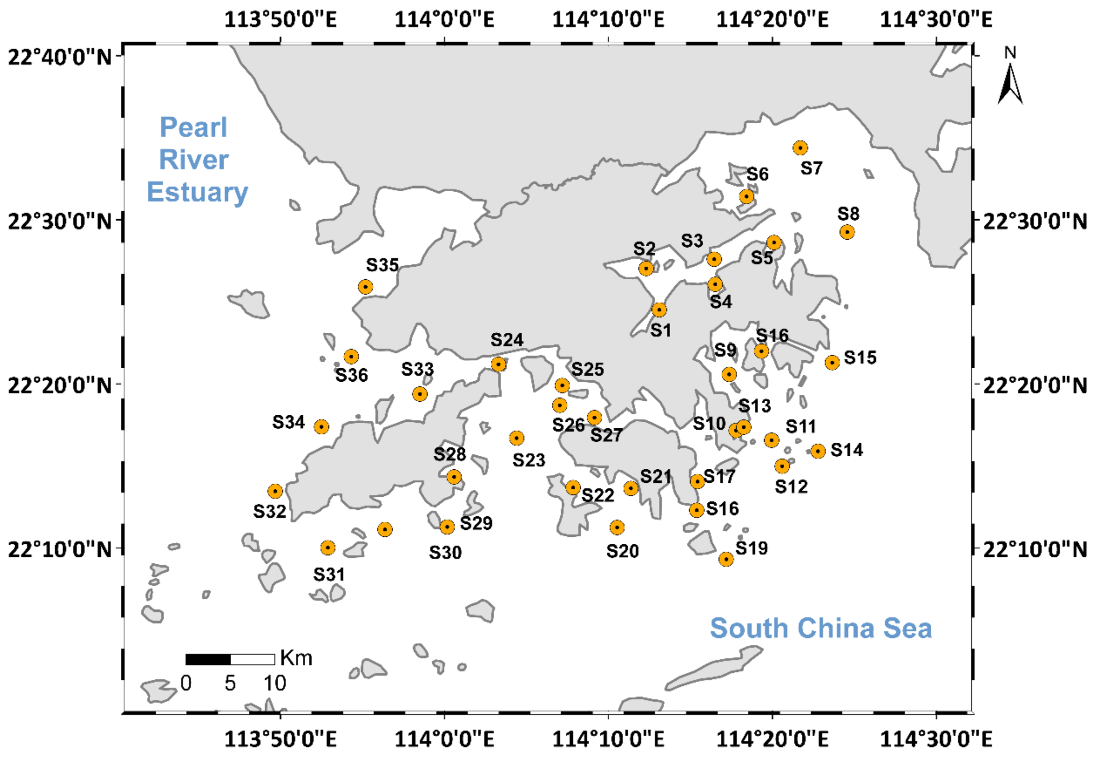

Figure S1 Sampling locations of surface seawater along the coastline of Hong Kong.

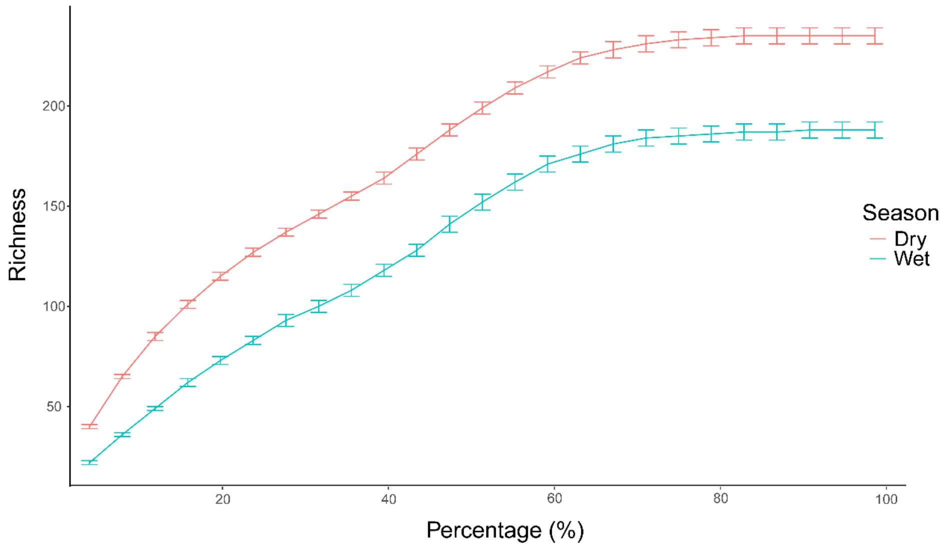

Figure S2 Rarefaction curves of the richness in the wet and dry seasons reach the saturation stage with means and standard errors under 1% - 100%, indicating that the number of samples in our study is sufficient to capture most microorganisms in each season.

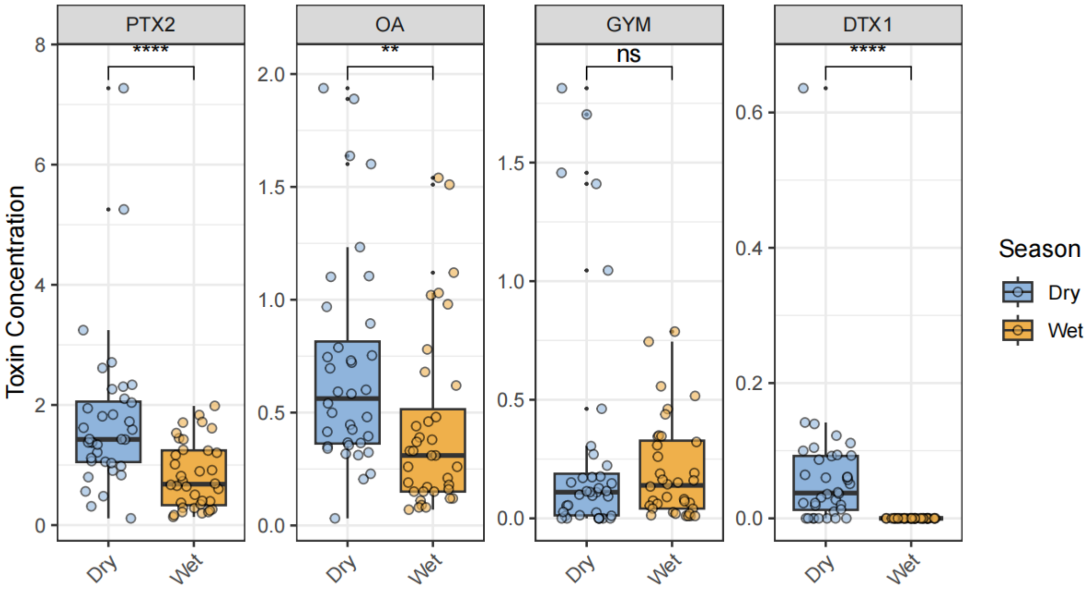

Figure S3 Seasonal patterns of targeted LATs.

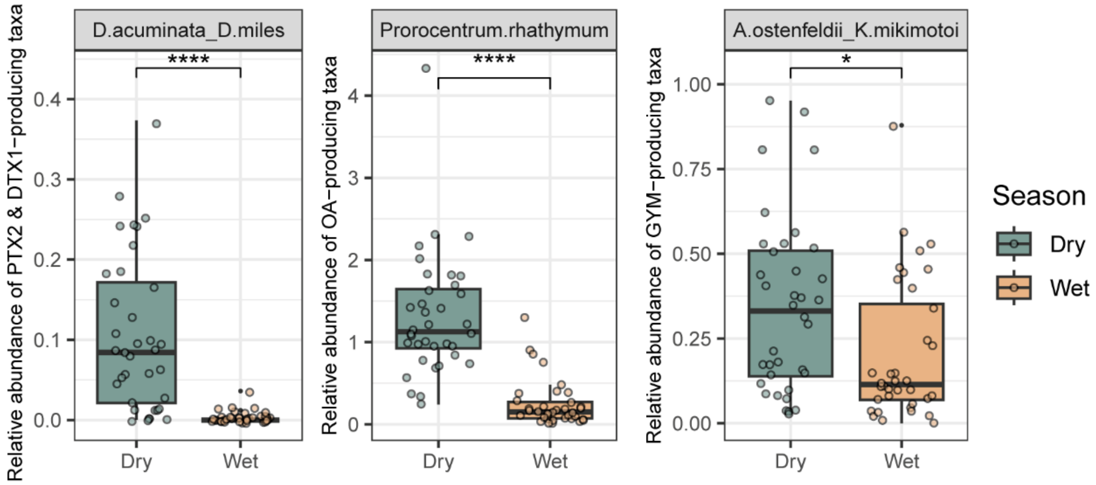

Figure S4 Seasonal patterns of potential producers.

References

1. Takahashi E, Yu Q, Eaglesham G, Connell DW, McBroom J, Costanzo S et al. Occurrence and seasonal variations of algal toxins in water, phytoplankton and shellfish from North Stradbroke Island, Queensland, Australia. *Mar Environ Res* 2007;64:429–42. 10.1016/j.marenvres.2007.03.005
2. Fux E, Gonzalez-Gil S, Lunven M, Gentien P, Hess P. Production of diarrhetic shellfish poisoning toxins and pectenotoxins at depths within and below the euphotic zone. *Toxicon* 2010;56:1487–96. 10.1016/j.toxicon.2010.09.007
3. Lee JS, Igarashi T, Fraga S, Dahl E, Hovgaard P, Yasumoto T. Determination of diarrhetic shellfish toxins in various dinoflagellate species. *J Appl Phycol* 1989;1:147–52. 10.1007/BF00003877
4. Kamiyama T, Suzuki T. Production of dinophysistoxin-1 and pectenotoxin-2 by a culture of *Dinophysis acuminata* (Dinophyceae). *Harmful Algae* 2009;8:312–7. 10.1016/j.hal.2008.07.003
5. Luisa FM, Reguera B, González-Gil S, Míguez A. Pectenotoxin-2 in single-cell isolates of *Dinophysis caudata* and *Dinophysis acuta* from the Galician Rías (NW Spain). *Toxicon* 2006;48:477–90. 10.1016/j.toxicon.2006.05.016
6. Marasigan AN, Sato S, Fukuyo Y, Kodama M. Accumulation of a high level of diarrhetic shellfish toxins in the green mussel *Perna viridis* during a bloom of *Dinophysis caudata* and *Dinophysis miles* in Sapian Bay, Panay Island, the Philippines. *Fish Sci* 2001;67:994–6. 10.1046/j.1444-2906.2001.00353.x
7. Giacobbe MG, Penna A, Ceredi A, Milandri A, Poletti R, Yang X. Toxicity and ribosomal DNA of the dinoflagellate *Dinophysis sacculus* (Dinophyta). *Phycologia* 2000;39:177–82. 10.2216/i0031-8884-39-3-177.1
8. Suzuki T, Miyazono A, Baba K, Sugawara R, Kamiyama T. LC–MS/MS analysis of okadaic acid analogues and other lipophilic toxins in single-cell isolates of several *Dinophysis* species collected in Hokkaido, Japan. *Harmful Algae* 2009;8:233–8. 10.1016/j.hal.2008.06.001
9. Murakami Y, Oshima Y, Yasumoto T. Identification of okadaic acid as a toxic component of a marine dinoflagellate *Prorocentrum lima*. *Nippon Suisan Gakkaishi* 1982;48:69–72. 10.2331/suisan.48.69
10. Morton SL. Morphology and toxicology of *Prorocentrum faustiae* sp. nov., a toxic species of non-planktonic dinoflagellate from Heron Island, Australia. *Bot. Mar.* 1998,41:565-70. 10.1515/botm.1998.41.1-6.565
11. Dickey RW, Bobzin SC, Faulkner DJ, Bencsath FA, Andrzejewski D. Identification of okadaic acid from a Caribbean dinoflagellate, *Prorocentrum concavum*. *Toxicon* 1990;28:371–7. 10.1016/0041-0101(90)90074-H
12. Morton SL, Bomber JW. Maximizing okadaic acid content from *Prorocentrum hoffmannianum* Faust. *J Appl Phycol* 1994;6:41–4. 10.1007/BF02185903
13. Zhou J, Fritz L. Ultrastructure of two toxic marine dinoflagellates, *Prorocentrum lima* and *Prorocentrum maculosum*. *Phycologia* 1993;32:444–50. 10.2216/i0031-8884-32-6-444.1
14. Morton SL, Moeller PDR, Young KA, Lanoue B. Okadaic acid production from the marine dinoflagellate *Prorocentrum belizeanum* Faust isolated from the

Belizean coral reef ecosystem. *Toxicon* 1998;36:201–6. 10.1016/S0041-0101(97)00054-8
15. Arteaga-Sogamoso E, Rodríguez F, Amato A, Ben-Gigirey B, Fraga S, Mafra LL et al. Morphology and phylogeny of *Prorocentrum porosum* sp. nov. (Dinophyceae): A new benthic toxic dinoflagellate from the Atlantic and Pacific Oceans. *Harmful Algae* 2023;121:102356. 10.1016/j.hal.2022.102356
16. Luo Z, Zhang H, Krock B, Lu S, Yang W, Gu H. Morphology, molecular phylogeny and okadaic acid production of epibenthic *Prorocentrum* (Dinophyceae) species from the northern South China Sea. *Algal Res* 2017;22:14–30. 10.1016/j.algal.2016.11.020
17. Cen J, Lu S, Moestrup Ø, Jiang T, Ho KC, Li S et al. Five *Karenia* species along the Chinese coast: With the description of a new species, *Karenia hui* sp. nov. (Kareniaceae, Dinophyta). *Harmful Algae* 2024;137:102645. 10.1016/j.hal.2024.102645
18. Van Wagoner RM, Misner I, Tomas CR, Wright JLC. Occurrence of 12-methylgymnodimine in a spirolide-producing dinoflagellate *Alexandrium peruvianum* and the biogenetic implications. *Tetrahedron Lett* 2011;52:4243–6. 10.1016/j.tetlet.2011.05.137
19. Van de Waal DB, Tillmann U, Martens H, Krock B, van Scheppingen Y, John U. Characterization of multiple isolates from an *Alexandrium ostenfeldii* bloom in The Netherlands. *Harmful Algae* 2015;49:94–104. 10.1016/j.hal.2015.08.002
